# Neurologic Adverse Events Associated With T-cell Engager Therapy in Multiple Myeloma: A Pharmacovigilance Study

**DOI:** 10.7759/cureus.87950

**Published:** 2025-07-14

**Authors:** Muhammad Atif Khan, Faiza Humayun Khan, Sohaib Irfan, Zahra Mahmoudjafari, Al-Ola Abdallah, Joseph P McGuirk, Nausheen Ahmed

**Affiliations:** 1 Internal Medicine, University of Kansas Medical Center, Kansas City, USA; 2 Internal Medicine, Montefiore St. Luke's Cornwall, Newburgh, USA; 3 Internal Medicine, Aga Khan University Hospital, Karachi, PAK; 4 Hematologic Malignancies and Cellular Therapeutics, University of Kansas Medical Center, Kansas City, USA

**Keywords:** elranatamab, neurological adverse events, talquetamab, tarlatamab, tce therapy

## Abstract

Introduction and aim

Relapse/refractory multiple myeloma (RRMM) presents significant therapeutic challenges despite recent advances. T-cell engager (TCE) therapies, such as teclistamab (TL), talquetamab (TQ), and elranatamab (ER), have emerged as promising options. However, these agents are associated with potentially life-threatening adverse effects (AEs), particularly neurological adverse effects (NAEs). This study aimed to assess the spectrum, reporting patterns, and relative likelihood of NAEs occurrence across TL, TQ, and ER.

Methods

We performed a retrospective analysis of the 2024 FDA Adverse Event Reporting System (FAERS) database using descriptive statistics and disproportionality analysis methods, including reporting odds ratio (ROR) and Empirical Bayes Geometric Mean (EBGM).

Results

Among the 1921 identified total AE cases, NAEs comprised 27.6% (530 cases), with TL accounting for the majority of the NAEs: 248 (46.79%) cases, followed by TQ: 201 (37.9%) cases, and ER: 81 (15.3%) cases. TQ demonstrated the greatest NAEs reporting proportion (RP) (39.2%), followed by TL (26.1%), and ER (17.6%). Among all reported NAEs, 159 events (26.9%) were immune effector cell-associated neurotoxicity syndrome (ICANS), while non-ICANS NAEs comprised 433 events (73.1%). ICANS were most frequently reported with TL (35.8% of NAEs), followed by ER (35.3%) and TQ (14%). TL also had the highest proportion of deaths among AE reports (24.4%). Disproportionality analysis revealed that TQ had the highest association with NAEs (ROR: 2.13; EBGM: 1.70), while ER had the lowest (ROR: 0.49; EBGM: 0.57). TL did not demonstrate a strong disproportionality signal (ROR: 0.85; EBGM: 0.92).

Conclusion

NAEs are a substantial part of the toxicity profile of TCEs in RRMM, particularly in the context of TL. These findings underscore the need for proactive neurologic monitoring and further research to identify predictive markers and improve management strategies.

## Introduction

Multiple myeloma (MM) is characterized by clonal proliferation of plasma cells, leading to the abnormal production of monoclonal immunoglobulins and specific end-organ damage [1]. Despite the availability of multiple first-line therapy options, MM remains incurable, and the development of relapse/refractory MM (RRMM) presents complex challenges for clinicians. However, novel therapies in RRMM, such as chimeric antigen receptor T cell (CAR-T) and T-cell engager (TCE) therapy, have led to an ongoing improvement in the five-year relative survival rate, which is currently 61.1%, according to the Surveillance, Epidemiology, and End Results (SEER) program from 2014 to 2020 [2-7]. TCE therapies function by binding simultaneously to a target antigen on myeloma cells, such as B-cell maturation antigen (BCMA) or G protein-coupled receptor family C group 5 member D (GPRC5D), and to CD3 receptors on T cells, thereby redirecting T cells to recognize and eliminate malignant plasma cells. TCE therapies targeting BCMA and GPRC5D, such as teclistamab (TL), talquetamab (TQ), and elranatamab (ER), have emerged as effective treatment options for patients with RRMM who have received at least four prior lines of therapy [8]. While a promising treatment approach, it is associated with potentially life-threatening toxicities including cytokine release syndrome (CRS), immune effector cell-associated neurotoxicity syndrome (ICANS), cytopenia(s), infections, hemophagocytic lymphohistiocytosis (HLH), and organ toxicity requiring close monitoring to mitigate potentially life-threatening complications [9].

Neurological adverse events (NAEs) are well described in CAR-T, such as ICANS and other neurological manifestations, including movement and neurocognitive treatment-emergent adverse events (MNTs), which comprise a cluster of movement disorders, cognitive changes, and personality changes, such as reduced facial expression and flat affect. The incidence of MNTs is as high as 5% with ciltacabtagene autoleucel, a BCMA-directed CAR-T, which is often insidious in onset and generally non-responsive to steroids [10]. Although ICANS is less common with TCE compared to BCMA-directed CAR-T therapy, with an incidence of approximately 3-3.4%, it can still significantly impact patients' quality of life [11,12]. However, the real-world characteristics and outcomes of NAEs are not well described.

We aimed to assess the spectrum and reporting patterns of NAEs related to TCE therapy in MM using the FDA Adverse Event Reporting System (FAERS) database. This includes analyzing reporting trends, comparing NAE profiles and mortality across TL, TQ, and ER, and evaluating the relative likelihood of NAE reporting for each therapy.

## Materials and methods

We conducted a retrospective analysis of adverse events (AEs) associated with the use of TCE therapy, including TL, TQ, and ER, in patients with MM, utilizing the 2024 FAERS. FAERS is a publicly available database that collects AEs submitted by health professionals (HP) or consumers. In the database, mortality, total AEs, and NAEs are reported at the case level (per patient), while individual neurological symptoms are recorded at the event level. Thus, one patient may report multiple neurologic symptoms, leading to more events than cases. The search was conducted in February 2025 and included all reports from the year 2024. We exclusively used data from the year 2024 to incorporate the most recent results and minimize bias related to over- or under-reporting due to varying FDA approval dates of the individual TCE therapies. The search terms used were talquetamab, talquetamab-tgvs, elranatamab, elranatamab-bcmm, teclistamab, and teclistamab-cqyv. The data were organized based on the source of the reports, which included HP and consumers, as well as the gender of the patients, categorized as male, female, or unknown. Age was excluded from the analysis due to inconsistencies and missing data. Descriptive statistical analysis was performed to calculate frequencies, proportions, and percentages. We calculated the proportion of each specific AE (e.g., ICANS) relative to the total number of NAEs reported for each drug, hereafter referred to as the reporting proportion (RP). AEs were classified using Medical Dictionary for Regulatory Activities (MedDRA)-preferred terms. NAEs were categorized into ICANS and non-ICANS subtypes. Since neurotoxicity (NT) has been reported independently of ICANS in the database, we classified it as non-ICANS. AEs with one or two events were excluded from the analysis. Taste disorders were grouped with neurological disorders in the database and were therefore included in the analysis. A comparative analysis was conducted to evaluate differences in NAE profiles across the three therapies - TL, TQ, and ER - by examining the reporting of specific event types and variations in reporting patterns.

Additionally, a disproportionality analysis was performed to assess the relationship among the three drugs and NAEs using the reporting odds ratio (ROR) and the Empirical Bayes Geometric Mean (EBGM). A 2×2 contingency table was constructed for each drug, comparing the number of reported NAEs to all other AEs for the drug and the other drugs combined. ROR was calculated as the odds of reporting a neurological event for a given drug compared to the other drugs, with statistical significance determined by 95% confidence intervals. EBGM and its credibility intervals (EB05, EB95) were estimated using Bayesian shrinkage techniques consistent with FDA Multi-Item Gamma Poisson Shrinker (MGPS) methodology to account for variability in reporting rates, particularly for rare events. Analyses were performed using R version 4.3.2 (Vienna, Austria: R Foundation for Statistical Computing). Cases with missing gender or reporter type were included, while events with only one to two reports were excluded to minimize the influence of outliers. As this study utilized publicly available, de-identified data, institutional review board (IRB) approval was not required.

## Results

Demographics for total adverse events

**Figure 1:**
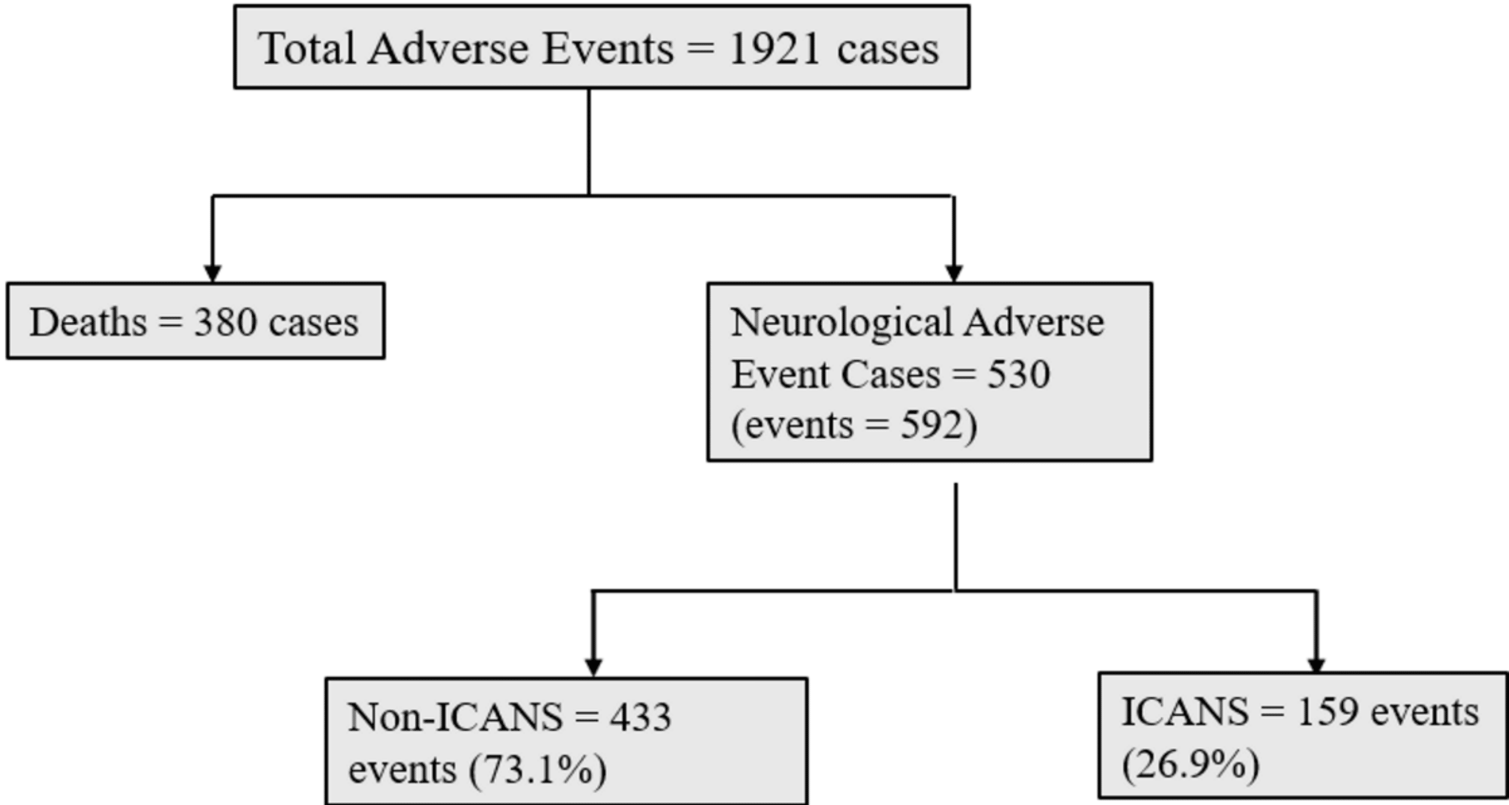
Flow chart representing classification of adverse event cases and events for T-cell engager therapy. ICANS: immune effector cell-associated neurotoxicity syndrome

A total of 1921 AEs were identified across three TCE therapies, TL (949 cases, 49.40%), TQ (513 cases, 26.70%), and ER (459 cases, 23.89%) (Figure 1 and Tables 1, 2). Among these, 683 (35.66%) reports were from male patients and 596 (31.02%) reported cases were from female patients, with 640 (33.32%) missing gender data (Table 1). The majority of reports came from HP: 1640 (85.37%), 275 (14.32%) were from consumers, and six (0.3%) had missing reporting sources (Table 1).

**Table 1:** Gender and reporting demographic for total cases of adverse events. HP: health professionals

Gender, n (%)	Teclistamab (n=949)	Talquetamab (n=513)	Elranatamab (n=459)	Total (n=1921)
Male	339 (35.72)	157 (30.60)	189 (41.18)	685 (35.66)
Female	303 (31.93)	113 (22.03)	180 (39.22)	596 (31.02)
Missing	307 (32.35)	243 (47.37)	90 (19.61)	640 (33.32)
Reporting, n (%)				
HP	834 (87.88)	385 (75.05)	421 (91.72)	1640 (85.37)
Consumer	111 (11.70)	126 (24.56)	38 (8.28)	275 (14.32)
Missing	4 (0.42)	2 (0.39)	-	6 (0.31)

**Table 2:** T-cell engager therapy mortality and adverse event cases profile. AE: adverse event; NAE: neurological adverse event

Variables	Teclistamab n (%)	Talquetamab n (%)	Elranatamab n (%)	Total
Total cases with AEs	949 (49.40)	513 (26.70)	459 (23.89)	1921
Total cases with NAEs	248 (46.79)	201 (37.92)	81 (15.28)	530
Total deaths	232 (61)	56 (14.74)	92 (24.21)	380
Deaths in patients with NAEs	57	13	12	82

Demographics for neurologic adverse events

Of the 1921 total AE cases, 530 (27.6%) were classified as NAEs. The distribution of these events according to therapy was as follows: TL (248 cases, 46.79%), TQ (201 cases, 37.92%), and ER (81 cases, 15.28%) (Figure 1 and Tables 2, 3). The gender distribution for NAEs showed 190 (35.58%) male and 159 (30%) female cases, with 181 (34.15%) reports missing gender data (Table 3). Similar to the total AEs, the majority of NAEs reports were from HP 398 (75.09%), 130 (24.53%) were from consumers, and two (0.38%) had missing reporting sources (Table 3).

**Table 3:** Gender and reporting demographic for total cases of neurologic adverse events. HP: health professionals

Gender, n (%)	Teclistamab (n=248)	Talquetamab (n=201)	Elranatamab (n=81)	Total (n=530)
Male	89 (35.89)	63 (31.34)	38 (46.91)	190 (35.85)
Female	80 (32.26)	51 (25.37)	28 (34.57)	159 (30.00)
Missing	79 (31.85)	87 (43.28)	15 (18.52)	181 (34.15)
Reporting, n (%)				
HP	197 (79.44)	126 (62.69)	75 (92.59)	398 (75.09)
Consumer	50 (20.16)	74 (36.82)	6 (7.41)	130 (24.53)
Missing	1 (0.40)	1 (0.50)	-	2 (0.38)

Outcomes

Mortality

A total of 380 deaths were reported as adverse outcomes, of which TL had 61.05% (232 cases), followed by ER with 24.21% (92 cases), and TQ with 14.74% (56 cases) (Figure 1 and Table 2). TL had the highest (24.4%) proportion (deaths/total AEs) of reported deaths, followed by ER (20%) and TQ (10.9%). Among individuals with NAEs, TL had the highest proportion (deaths/NAEs) mortality reported (22.9%), followed by ER (14.8%) and TQ (6.4%).

Neurologic Adverse Events

The analysis showed that TQ had the highest association with NAEs, with ROR of 2.13 (95% CI: 1.72-2.65) and an EBGM of 1.70 (95% CI: 1.37-2.11). ER was the least associated, with ROR of 0.49 (95% CI: 0.37-0.64) and an EBGM of 0.57 (95% CI: 0.43-0.74), indicating a lower risk of NAE compared to the other drugs. TL did not have a strong signal for disproportionality reporting, with ROR of 0.85 (95% CI: 0.70-1.04) and an EBGM of 0.92 (95% CI: 0.75-1.13), implying a similar reporting rate to the other drugs (Table 4).

**Table 4:** Reporting odds ratio and EBGM of all TCE therapies. ROR: reporting odds ratio; EBGM: Empirical Bayes Geometric Mean; CI: confidence interval; TCE: T-cell engager

Drug	ROR, 95% CI	EBGM, 95% CI
Teclistamab	0.85 (0.70-1.04)	0.92 (0.75-1.13)
Talquetamab	2.13 (1.72-2.65)	1.70 (1.37-2.11)
Elranatamab	0.49 (0.37-0.64)	0.57 (0.43-0.74)

A total of 530 cases reported NAEs, while the number of individual NAEs was 592, as some patients experienced and reported multiple neurologic symptoms, resulting in a higher event count than case count (Figure 1). TQ exhibited the highest RP of NAEs at 39.2%, compared to TL at 26.1% and ER at 17.6%. Out of 592 NAEs, 159 events (25.9%) were ICANS and 433 events (73.1%) were non-ICANS. TL had the highest proportion of ICANS reports (35.8%), closely followed by ER (35.3%), with a lower proportion observed for TQ (14%) (Table 5). Among non-ICANS NAEs, TQ had the highest RP at 86%, followed by ER (64.7%) and TL (64.2%), which could be partly explained by the taste disorders. TQ, as expected due to its mechanism of action, had the highest RP for taste disorder (62.4%), followed by TL (3.4%) and ER (1.1%). Peripheral neuropathy had RP of 14.1% in ER, 8.7% in TL, and 3.7% in TQ (Table 5).

**Table 5:** Total number of neurological adverse events reported for each drug. ^*^Reporting proportion. NAE: neurological adverse event; AE: adverse event; ICANS: immune effector cell-associated neurotoxicity syndrome; CVA: cerebrovascular accident; LOC: loss of consciousness; GBS: Guillain-Barré syndrome

Variables	Teclistamab (n=265), n (%)^*^	Talquetamab (n=242), n (%)^*^	Elranatamab (n=85), n (%)^*^	Total events (n=592), n
ICANS NAEs	95 (35.8)	34 (14)	30 (35.3)	159
Non-ICANS NAEs	170 (64.2)	208 (86)	55 (64.7)	433
Taste disorder	9 (3.4)	151 (62.4)	1 (1.1)	161
Peripheral neuropathy	23 (8.7)	9 (3.7)	12 (14.1)	44
Neurotoxicity	18 (6.8)	3 (1.2)	6 (7)	27
Headache	17	5	3	25
Depressed or altered level of consciousness	14	2	7	23
Disturbance in attention	12	2	na	14
Dizziness	11	6	na	17
Tremor	9	6	4	19
Seizure	6	1	2	9
Cognitive disorder	11	2	3	16
CVA	5	4	2	11
LOC or syncope	7	1	1	9
Restless leg syndrome	2	na	na	2
Paresthesia	5	5	1	11
GBS	1	1	1	3
Anosmia/paraosmia	na	5	1	6
Cranial nerve palsy	2	1	6	9
Encephalopathy	6	2	2	10
Somnolence/lethargy	12	2	3	17

Headache was also common, with a total of 25 events reported, primarily in TL (17 events). Depressed or altered level of consciousness had 23 events, with major contribution from TL (14 events). Other infrequent NAEs included seizures (nine events), the majority contributed by TL (six events), and tremors (19 events), which were most frequent in TL (nine events) and TQ (six events). Guillain-Barré syndrome (GBS) (three events) and cranial nerve palsies (nine events), though serious, were less frequently reported (Table 5). Out of nine cranial nerve palsies, seven were facial palsies (five in ER and two in TL).

## Discussion

NAEs accounted for 27.6% of all AEs in our analysis, notably higher than the RP NAEs in pivotal studies of TQ (21.6%) and TL (14.5%) [12,13]. However, while the lack of total patient numbers in our study prevents direct incidence rate comparisons, the substantial proportion of AEs being neurologic highlights their significant impact. This can impact a patient's quality of life; therefore, we emphasize the importance of vigilance in counseling patients about symptoms, as well as identifying and managing NAEs early to mitigate the risk of deterioration. TQ had the highest association with NAEs reporting, with ROR of 2.13 (95% CI: 1.72-2.65) and an EBGM of 1.70 (95% CI: 1.37-2.11), while ER showed a lower, and TL showed almost a similar reporting rate as compared to others. This is a notable finding because the incidence of NAEs in the pivotal TQ study has remained slightly lower than TL (10% vs. 14.5%) [12,13]. This may be attributed to GPRC5D receptor engagement by TQ, in contrast to BCMA targeting by the other agents, which likely contributes to the disproportionately higher RP of taste disorders observed in the TQ group. Our analysis across the three TCE therapies revealed notable differences in both the total AEs and NAEs. The distribution of AEs revealed that TL was associated with the largest share of both total AEs (49.40%) and neurological AEs (46.79%), indicating a higher reporting of AEs than TQ (26.70% for total AEs, 37.92% for NAEs) and ER (23.89% for total AEs, 15.28% for NAEs). The predominance of adverse events with TL may be attributed to its earlier FDA approval in 2022, allowing for broader use and greater physician familiarity and confidence in prescribing it compared to ER and TQ, both approved in 2023 [5-7]. Alternatively, it may reflect a higher toxicity profile, underscoring the need for vigilant monitoring and early intervention in clinical settings to mitigate the risk of poor outcomes [5].

Among NAEs, ICANS was one of the most frequently reported events across all therapies, with the highest RP observed in TL (35.8%), followed by ER (35.3%) and TQ (14%). Peripheral neuropathy and NT were also commonly reported, with the highest RP seen in ER (14.1% and 7%, respectively), followed by TL (8.7% and 6.8%, respectively), which further emphasizes the need for careful neurologic assessment during its use. This differs from almost similar incidence of ICANS reported by Lesokhin et al. (3.4%) and Moreau et al. (3%) [11,12]. Given the higher reporting rate in TL, it is crucial for clinicians to closely monitor patients for early signs of neurotoxicity, particularly in patients with pre-existing neurological conditions. Strategies, such as dose adjustment, temporary discontinuation, or the use of corticosteroids for managing ICANS, may be necessary to mitigate these effects [12,14]. In contrast, ER had a lower NAEs RP, which may reflect its distinct pharmacologic properties or lower patient exposure in the FAERS data. Despite this, ER still contributed to a significant proportion of the mortality, representing 20% RP, second only to TL (24.4%). Of the nine reported nerve palsies, seven were cases of facial nerve paralysis, with the majority occurring in the ER group (five events).

Furthermore, the identification of taste disorders as a commonly reported NAE in the FAERS database, with major contribution from TQ, suggests a potential impact on patients' quality of life. This underscores the need for a comprehensive evaluation of the overall toxicity profile of these therapies. The higher RP of taste disorders in TQ aligns with findings by Chari et al., who observed such disorders in 57-63% of patients experiencing adverse events [13]. As reported by Chari et al., the occurrence of taste disorders may be linked to the GPRC5D receptor expression in the filiform papillae of the tongue [13]. TQ targets this receptor to mediate the elimination of GPRC5D-expressing myeloma cells, which may explain its role in inducing taste disturbances [13,15]. Monitoring and managing NAEs are essential for enhancing the safety and tolerance of TCE therapy, preventing interruptions or discontinuations of an otherwise effective treatment.

Our study had limitations that may affect the interpretation of these findings. First, the database relies on voluntary reporting, which may result in underreporting or incomplete data. Additionally, the absence of total patient numbers prevents the determination of the incidence of AEs; hence, relying on the reporting rate limits the interpretation of ROR as indicators of reporting trends instead of risk. Second, detailed information regarding comorbidities and AEs is lacking, including AE grade, time to onset, duration until resolution, and management approaches. Furthermore, the database does not offer precise characterization of specific AEs, such as neurotoxicity. These limitations highlight the need for caution when generalizing the results and emphasize the importance of further controlled studies to confirm these findings. However, it plays a crucial role in post-marketing surveillance by providing real-world data on the safety of TCE therapy after its approval. This allows for the detection of rare or long-term side effects that may only emerge after a drug is used in larger and more diverse populations.

## Conclusions

This study examined the reporting patterns of NAEs associated with BCMA and GPRC5D TCE therapy in RRMM. Our data offers insights into the spectrum of neurologic toxicities associated with TCE in RRMM, revealing a significantly higher RP of ICANS and non-ICANS NAEs (excluding taste disorders) with TL compared to TQ and ER. Notably, taste disorders are frequently observed with TQ. Further prospective trials are required to identify predictive baseline characteristics by imaging studies or biomarkers that enable early intervention to mitigate NAEs. To optimize treatment outcomes, new strategies must be developed to identify individuals at higher risk, particularly those with pre-existing neurologic conditions.
